# Cooperation of hydrolysis modes among xylanases reveals the mechanism of hemicellulose hydrolysis by *Penicillium chrysogenum* P33

**DOI:** 10.1186/s12934-019-1212-z

**Published:** 2019-09-21

**Authors:** Yi Yang, Jinshui Yang, Ruonan Wang, Jiawen Liu, Yu Zhang, Liang Liu, Fengqin Wang, Hongli Yuan

**Affiliations:** 1 State Key Laboratory of Agrobiotechnology, College of Biological Sciences, China Agricultural University, Beijing, 100193 China; 2 College of Forestry, Henan Agricultural University, Zhengzhou, 450002 China; 3 College of Life Science, Henan Agricultural University, Zhengzhou, 450002 China

**Keywords:** *Penicillium chrysogenum*, Xylanase, Enzymatic hydrolysis, Cooperation, Hydrolysis mode

## Abstract

**Background:**

Xylanases randomly cleave the internal β-1,4-glycosidic bonds in the xylan backbone and are grouped into different families in the carbohydrate-active enzyme (CAZy) database. Although multiple xylanases are detected in single strains of many filamentous fungi, no study has been reported on the composition, synergistic effect, and mode of action in a complete set of xylanases secreted by the same microorganism.

**Results:**

All three xylanases secreted by *Penicillium chrysogenum* P33 were expressed and characterized. The enzymes Xyl1 and Xyl3 belong to the GH10 family and Xyl3 contains a CBM1 domain at its C-terminal, whereas Xyl2 belongs to the GH11 family. The optimal temperature/pH values were 35 °C/6.0, 50 °C/5.0 and 55 °C/6.0 for Xyl1, Xyl2, and Xyl3, respectively. The three xylanases exhibited synergistic effects, with the maximum synergy observed between Xyl3 and Xyl2, which are from different families. The synergy between xylanases could also improve the hydrolysis of cellulase (C), with the maximum amount of reducing sugars (5.68 mg/mL) observed using the combination of C + Xyl2 + Xyl3. Although the enzymatic activity of Xyl1 toward xylan was low, it was shown to be capable of hydrolyzing xylooligosaccharides into xylose. Xyl2 was shown to hydrolyze xylan to long-chain xylooligosaccharides, whereas Xyl3 hydrolyzed xylan to xylooligosaccharides with a lower degree of polymerization.

**Conclusions:**

Synergistic effect exists among different xylanases, and it was higher between xylanases from different families. The cooperation of hydrolysis modes comprised the primary mechanism for the observed synergy between different xylanases. This study demonstrated, for the first time, that the hydrolysates of GH11 xylanases can be further hydrolyzed by GH10 xylanases, but not vice versa.

## Background

As the second most abundant polysaccharide on earth, hemicellulose is composed of β-1,4-linked d-xylopyranosyl residues with side chains that contain various substituents, such as arabinose, galactose, and acetyl groups [1]. The hydrolysis of hemicellulose, especially xylan, is considered to be a limiting factor in cellulose degradation [2, 3]. Xylanases randomly cleave the internal β-1,4-glycosidic bonds in the xylan backbone and are grouped into the glycoside hydrolase (GH) families 5, 8, 10, 11, 30, and 43 in the carbohydrate-active enzyme (CAZy) database (http://www.cazy.org), with most belonging to GH10 and GH11 [4]. Some xylanases also contain carbohydrate-binding modules (CBMs), which play important roles in the binding of enzymes to substrates [5]. Various substrate specificities and catalytic activities exist among the different families of xylanases. GH10 xylanases exhibit low substrate specificity and have high affinity toward the highly branched xylan backbone [6]. In addition, these enzymes have small substrate binding sites that have a high affinity toward shorter linear xylooligosaccharides [7, 8]. In contrast, GH11 xylanases require at least three consecutive unsubstituted xylose residues and cannot cleave glycosidic linkages next to a branch [9]; thus, they prefer to hydrolyze long chain xylooligosaccharides [10]. GH11 xylanases have been recognized as “true” xylanases because of their exclusive activity toward D-xylose containing substrates [11, 12].

The structure and composition of xylan in lignocellulosic materials is known to be complex, and the xylosidic linkages are not equally accessible to xylanases [13, 14]. Therefore, multiple xylanases with different cleavage patterns of xylosidic bonds are required to increase the extent of xylan hydrolysis to monosaccharide [15]. For this reason, many filamentous fungi may produce different xylanases in the presence of lignocellulosic materials. For example, *Myceliophthora thermophila* produces three GH10 and five GH11 xylanases [11, 16, 17], *Fusarium graminearum* produces two GH10 and two GH11 xylanases [18], *Aspergillus fumigatus* produces three GH10 and one GH11 xylanases [19], and *Penicillium oxalicum* produces two GH10, five GH11 and one GH30 xylanases [20]. Thus, harboring multiple xylanases with different specific functions that are produced in the presence of lignocellulose may be a strategy used by microorganisms to promote efficient xylan hydrolysis [13, 14, 21]. However, until now, the composition, synergistic effect, and mode of action of a complete set of xylanases secreted by a single microorganism under optimum inducing conditions have not been explored. Therefore, the synergistic mechanism by which different xylanases from the same microorganism promote the degradation of xylan is unclear and remains to be elucidated. An understanding of this process may reveal the mechanism of hemicellulose degradation by microorganisms and show their adaptability to the natural environment.

In our previous study, an enzyme cocktail that was primarily composed of hemicellulases was described in *Penicillium chrysogenum* P33 and was shown to significantly enhance the hydrolytic performance of commercial cellulase against various lignocellulosic biomass [22]. In addition, the results of a secretome analysis showed that in the presence of wheat bran plus microcrystalline cellulose, P33 secreted three xylanases, including two GH10 family xylanases and a GH11 family xylanase, with one of the GH10 xylanases containing a CBM1 domain at its C-terminus [22]. In this study, all three xylanases from P33 were expressed in *Pichia pastoris* GS115. The enzymatic characteristics, synergism, and ability to promote the hydrolysis of cellulase were studied. Furthermore, the hydrolysis modes of the three enzymes were determined to elucidate the mechanism of the observed synergism. The results of this study show the mechanism by which *P. chrysogenum* P33 degrades xylan in lignocellulosic biomass in nature and provide a basis for designing efficient enzyme systems.

## Methods

### Strains, media, vectors and chemicals

*Escherichia coli* DH5α was purchased from Biomed (Beijing, China) and grown in Luria-Bertani (LB) medium at 37 °C for gene cloning. *Pichia pastoris* GS115 was purchased from Invitrogen (MA, USA) and cultivated in yeast peptone dextrose (YPD) medium at 28 °C for use as the host strain for gene expression following the guidelines of the *Pichia* expression system manual (Invitrogen). The vector pPIC9 K (Invitrogen) was used for xylanase expression in *P. pastoris*. A DNA purification kit was purchased from Tiangen (Beijing, China), and T4 DNA ligase was purchased from Promega (Madison, USA). Phusion DNA polymerase and restriction endonucleases were purchased from New England Biolabs (MA, USA). Beechwood xylan (Lot#170728) was purchased from Megazyme (Wicklow, Ireland) and used as a substrate. Commercial cellulase from *Trichoderma longibrachiatum* was purchased from Sigma (C9748) with 12.9% protein content and 19.7 U/mg endoglucanase activity. Delignified corn stover was prepared as described previously [23]. All other chemicals used in this study were of analytical grade and are commercially available.

### Construction of the recombinant plasmids and heterologous expression in *P. pastoris*

The xylanase-encoding genes, excluding their signal sequences, were PCR amplified using the P33 cDNA as template and the specific primers Xyl1F′ and Xyl1R′, Xyl2F′ and Xyl2R′, and Xyl3F′ and Xyl3R′ (Additional file 1: Table S1). The constructed recombinant plasmids were linearized with *Sal*I (New England BioLabs) and then transformed into *P. pastoris* GS115 by electroporation according to the manufacturer’s instructions. The transformed cells were spread onto MD agar plates and incubated at 28 °C for 3–4 days, and the resulting transformants were subsequently spread onto YPD agar plates containing different concentrations of G418 (geneticin). The transformants carrying the target genes were identified by PCR, and the level of protein expression in the recombinant transformants was validated in BMMY medium with 2% (v/v) methanol as an inducer.

### Purification of the recombinant xylanases

The extracellular protein content of *P. pastoris* increased gradually with the induction time until 72 h, and then decreased. Therefore, after 72 h of induction, the cell-free supernatant of each *P. pastoris* culture was collected by centrifugation at 4 °C, 8000 rpm for 10 min and then filtered through a 0.45-μm filter. Next, the supernatant was loaded onto a Ni^2+^ His-tag column (GE healthcare, USA) that was equilibrated with binding buffer (20 mM sodium phosphate and 0.5 M NaCl, pH 7.4). The nonspecific-binding proteins were removed by washing with buffer (20 mM sodium phosphate, 0.5 M NaCl, and 20 mM imidazole, pH 7.4) and the bound protein was eluted with elution buffer (20 mM sodium phosphate, 0.5 M NaCl, and 300 mM imidazole, pH 7.4).

### Biochemical characterization of the purified recombinant xylanases

The xylanase activity of the purified enzymes was determined using the dinitrosalicylic acid (DNS) method [24] with 1.0% (w/v) beechwood xylan as a substrate. One unit of xylanase activity was defined as the amount of enzyme required to release 1 μmol of xylose per min under the assay conditions. The optimal pH for xylanase activity was determined at 40 °C using citric acid-Na_2_HPO_4_ buffer with pH values ranging from pH 3.0–8.5. The optimal temperature was determined in the range of 10–90 °C at the optimal pH value. The effect of different metal ions and chemical reagents on the xylanase activity was determined in the presence of 10 mM Ca^2+^, K^+^, Ag^+^, Pb^2+^, Ni^2+^, Mg^2+^, Cu^2+^, Co^2+^, Na^+^, Fe^3+^, Zn^2+^ or Al^3+^, with an enzyme reaction without any added metal ions used as a control. The relative enzyme activity was calculated using the activity of the enzyme without any added metal ions as 100%. The kinetic parameters of the enzymes were assessed using the Michaelis–Menten equation with GraphPad Prism 5 by applying nonlinear regression [25]. All of the experiments were performed in duplicate under standard conditions.

### Hydrolytic properties of the purified xylanases

The hydrolytic products of xylan and xylooligosaccharides produced by the purified xylanases were analyzed via thin-layer chromatography (TLC). The purified xylanases (400 ng) were incubated with 0.5% beechwood xylan or 0.2% xylooligosaccharides in sodium hydrogen phosphate-citric acid buffer (pH 5.5) in a final volume of 5 μL at 45 °C for 24 h. Sequential hydrolysis was conducted by incubating beechwood xylan with each xylanase individually for 2 h. Subsequently, the three xylanases were individually added to the prehydrolyzed mixture and then incubated for another 4 h. After hydrolyzing for the indicated time, hydrolysis was terminated by boiling the reaction mixture at 100 °C for 30 min to inactivate the enzymes and then centrifuged at 12,000 rpm for 10 min. The hydrolytic products were spotted onto silica gel plates (60F254; Merk, Darmstadt, Germany). The plates were developed using a butanol:acetic acid:water (5:4:1, v/v/v) solvent system. After spraying the plates with a methanol-sulfuric acid mixture (4:1, v/v), the color reaction was performed in an oven at 105 °C for 10 min. Xylose (X_1_), xylobiose (X_2_), xylotriose (X_3_), xylotetraose (X_4_) and xylopentaose (X_5_) were purchased from Megazyme (Wicklow, Ireland) and used as standards.

To determine the amount of products released by Xyl1 and Xyl3 from xylooligosaccharides, 400 ng of purified Xyl1 or Xyl3 were incubated with 0.2% (w/v) xylooligosaccharides (from X_3_ to X_5_) in sodium hydrogen phosphate-citric acid buffer (pH 6.0) in a final volume of 30 μL at 35 °C (Xyl1) or 55 °C (Xyl3) for 1 h. After that, the reaction mixture was boiled at 100 °C for 30 min and then centrifuged at 12,000 rpm for 10 min. The amount of released xylooligosaccharides was measured by high-performance liquid chromatography (HPLC) using a previously described method [26].

### Enzymatic hydrolysis

Hydrolysis experiments were conducted with 2% (w/v) dried delignified corn stover in a final reaction volume of 1 mL [26]. Delignified corn stover was composed of 45.96% glucan, 20.40% xylan, and 9.93% lignin. For the hydrolysis assays using the xylanases individually, the experiments were conducted in 50 mM sodium acetate buffer (pH 5.5) using 5 mg protein/g glucan in an orbital shaker incubator at 45 °C. For the synergistic hydrolysis of xylanase and commercial cellulase, reactions were performed in 50 mM sodium acetate buffer (pH 5.0) in an orbital shaker incubator at 37 °C. Commercial cellulase was assessed using 10 mg protein/g glucan. The individual xylanases were assessed using 5 mg protein/g glucan. A protein ratio of 1:1 and 1:1:1 were used when two xylanases and three xylanases were simultaneously added, respectively, with the total xylanase added maintained at 5 mg protein/g glucan. An enzyme reaction without substrate and substrate without enzyme treatments were included under the same conditions as controls.

After incubating for the indicated time, hydrolysis was terminated by boiling the reaction mixture at 100 °C for 30 min to inactivate the enzymes. The supernatants were collected by centrifugation at 12,000 rpm for 10 min and were used for further analyses. The concentration of total reducing sugars was determined using the DNS method [24]. The individual contents of glucose, cellobiose and xylose were measured by HPLC as described previously [26]. All hydrolysis assays were performed in triplicate, and the mean values and standard deviations are presented.

The glucan and xylan conversions during the hydrolysis were calculated according to the following equations, respectively:

$$~{\text{Glucan conversion }}\left( \%  \right){\text{ }} = \frac{{\left( {{{\text{C}}_{{\text{cellobiose}}}} \times ~0.947~ + ~{{\text{C}}_{{\text{glucose}}}}~ \times ~0.9} \right)}}{{{{\text{M}}_{{\text{substrate}}}}~ \times ~{\text{glucan~content~in~the~delignified~corn~stover~}}}}~ \times 100$$

$$~{\text{Xylan conversion }}\left( \%  \right){\text{ }} = \frac{{{{\text{C}}_{{\text{xylose}}}}{\text{~}} \times ~0.88}}{{{{\text{M}}_{{\text{substrate}}}}{\text{~}} \times ~{\text{xylan~content~in~the~delignified~corn~stover}}~}}~ \times 100$$

where C_cellobiose_, C_glucose_, and C_xylose_ indicate the concentration of cellobiose, glucose, and xylose in the hydrolysate, respectively; M_substrate_ indicates the substrate (delignified corn stover) loading.

The degree of synergism (DS) between different enzymes was calculated as the following equation:

$$ {\text{DS }} = \frac{\text{A1 + 2}}{{ ( {\text{A1 + A2)}}}} $$

where A_1+2_ indicates the amount of reducing sugars generated by the combination of all the enzymes in one hydrolysis reaction; A_1_ and A_2_ indicate the amount of reducing sugars achieved with each enzyme in individual hydrolysis assays.

Statistical significance was assessed by analysis of variance (ANOVA, Duncan test), with a *p* value < 0.05 deemed significant.

## Results

### Gene cloning and sequence analysis

**Fig. 1:**
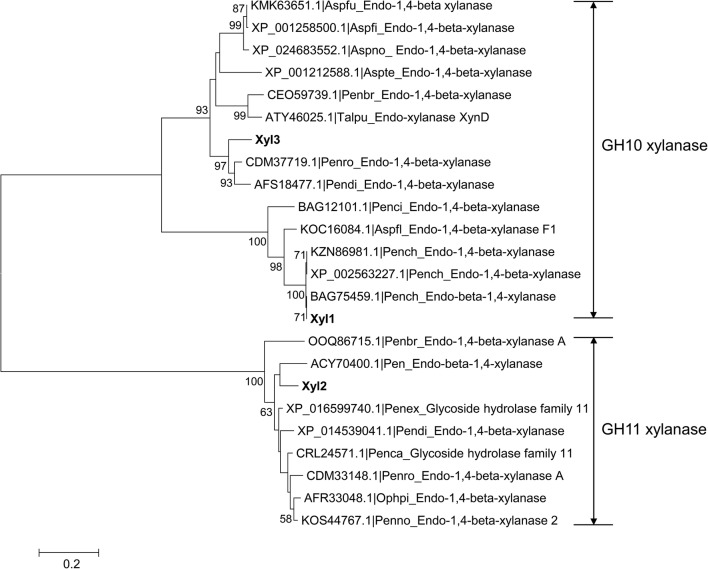
Phylogenetic tree resulting from the analysis of amino acid sequences of the three xylanases and other xylanases using the Neighbor-Joining method. The numbers on nodes correspond to the percentage bootstrap values for 1000 replicates and only the values higher than 50% were shown. Aspfu: *Aspergillus fumigatus*; Aspfi: *Aspergillus fischeri*; Aspno: *Aspergillus novofumigatus*; Aspte: *Aspergillus terreus*; Penbr: *Penicillium brasilianum*; Talpu: *Talaromyces purpureogenus*; Penro: *Penicillium roqueforti*; Pendi: *Penicillium digitatum*; Penci: *Penicillium citrinum*; Aspfl: *Aspergillus flavus*; Pench: *Penicillium chrysogenum*; Pen: *Penicillium* sp.; Penex: *Penicillium expansum*; Penca: *Penicillium camemberti*; Ophpi: *Ophiostoma piliferum*; Penno: *Penicillium nordicum*

**Fig. 2:**
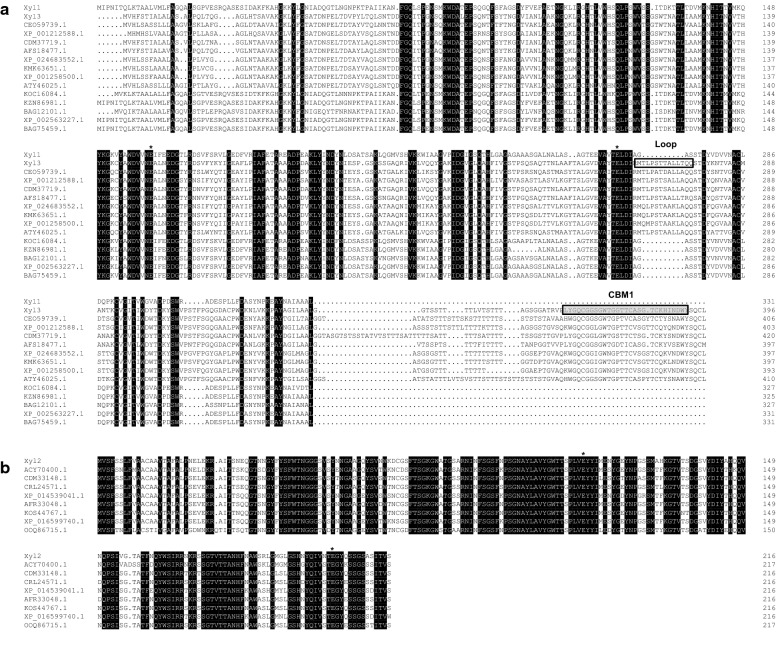
Alignment of P33 xylanases with different heterologous xylanases. **a** Alignment of Xyl1 and Xyl3 with different GH10 xylanase; **b** Alignment of Xyl2 with different GH11 xylanases. The catalytic residues were marked with *, the residues in the gray colored boxes are the CBM1 residues and the residues in the box is the loop of Xyl3

The genes of all the three xylanases secreted by *P. chrysogenum* P33 were successfully cloned and sequenced. The full-length cDNA of *xyl1* (GenBank accession number: MF960910), *xyl2* (GenBank accession number: MF960911) and *xyl3* (GenBank accession number: MF960912) were 996 bp, 651 bp and 1191 bp in length, corresponding to proteins composed of 331, 216 and 396 amino acids, respectively. The amino acid alignments of Xyl1, Xyl2 and Xyl3 showed 100%, 100% and 99% identity with the endo-β-1,4-xylanase from *P. chrysogenum* (Accession number: BAG75459.1), and the proteins Pc12g01520 (Accession number: XP_002557047.1) and Pc22g00820 (Accession number: XP_002564127.1) from *P. rubens* Wisconsin 54-1255, respectively. Xyl1 showed 10% and 46% amino acid sequence identity to Xyl2 and Xyl3, respectively, while Xyl2 shared 36% sequence identity to Xyl3. In the phylogenetic tree reconstructed using the neighbor-joining method (Fig. 1), Xyl1 and Xyl3 were grouped into the GH10 family, whereas Xyl2 was grouped into the GH11 family according to the domain annotation from CAZy, which confirmed the previously reported results [22]. Amino acid residues 1-23, 1-19 and 1-21 were identified as the signal peptides of Xyl1, Xyl2 and Xyl3, respectively, suggesting that they are all extracellular proteins. Alignments of the three xylanases and their reference sequences revealed that Glu161 and Glu267, Glu112 and Glu203, and Glu152 and Glu259 are conserved catalytic residues of Xyl1, Xyl2 and Xyl3, respectively (Fig. 2). The results of the conserved domain search showed that Xyl3 also contains a CBM1 domain. Furthermore, although both Xyl1 and Xyl3 are GH10 xylanases, Xyl3 possesses an extra loop compared with Xyl1 (Fig. 2).

### Expression and purification of the recombinant xylanases

**Fig. 3:**
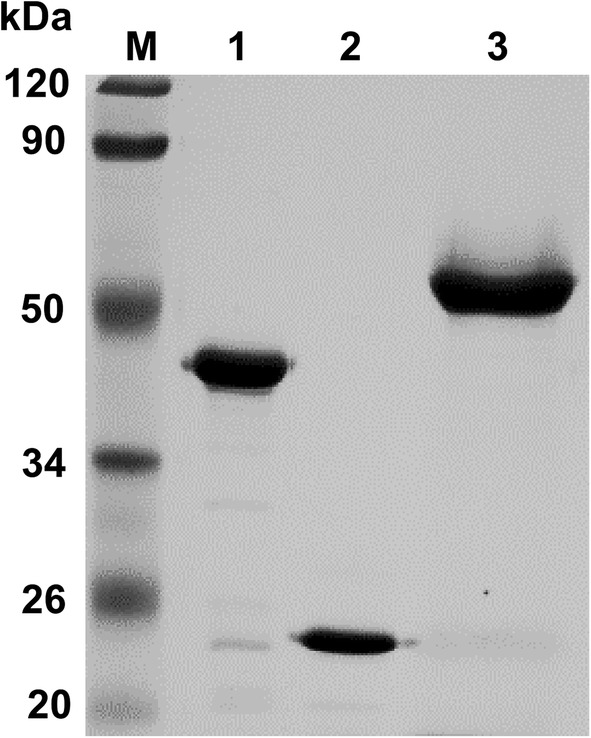
SDS-PAGE analysis of purified Xyl1, Xyl2 and Xyl3. Lanes: M, standard protein molecular weight markers; 1, Xyl1; 2, Xyl2; 3, Xyl3

The gene fragments encoding the mature Xyl1, Xyl2, and Xyl3 enzymes without signal peptides were successfully expressed in *P. pastoris* GS115. All of the recombinant xylanases were subsequently purified to homogeneity according to SDS-PAGE, and the apparent molecular weights were estimated to be 42.7 kDa, 23.5 kDa, and 52.7 kDa for Xyl1, Xyl2, and Xyl3, respectively (Fig. 3).

### Enzymatic properties of the recombinant xylanases

Using 1% (w/v) beechwood xylan as a substrate, the specific activities of Xyl1, Xyl2, and Xyl3 were determined to be 151.5 U/mg, 2149.8 U/mg, and 290.6 U/mg, respectively (Table 1). Xyl1 showed a *V*_max_ value of 327.2 μmol/min/mg and a *k*_cat_/*K*_m_ value of 18.7 mL/mg/s. The *V*_max_ and *k*_cat_/*K*_m_ values of Xyl3 were 392.4 μmol/min/mg and 54.7 mL/mg/s, respectively. The catalytic efficiency (*k*_cat_/*K*_m_) of Xyl2 was 260.5 mL/mg/s, which was 13.9- and 4.8-fold greater than that of Xyl1 and Xyl3, respectively.

**Table 1:** The specific activities and kinetic parameters of the purified recombinant xylanases

Enzymes	Specific activity (U/mg)	*V*_max_ (μmol/min/mg)	*K*_m_ (mg/mL)	*k*_cat_ (s^−1^)	*k*_cat_/*K*_m_ (mL/mg/s)
Xyl1	151.5 ± 4.7	327.2 ± 13.29	9.6 ± 1.2	180.2 ± 7.3	18.7 ± 0.8
Xyl2	2149.8 ± 43.1	2779 ± 101.5	3.8 ± 0.5	990.7 ± 36.2	260.5 ± 9.6
Xyl3	290.6 ± 7.9	392.4 ± 51.08	4.8 ± 1.4	262.1 ± 34.1	54.7 ± 7.1

Reactions were performed at each corresponding optimal conditions

The experiments were performed in duplicate, and the data are presented as the means ± standard deviations

**Fig. 4:**
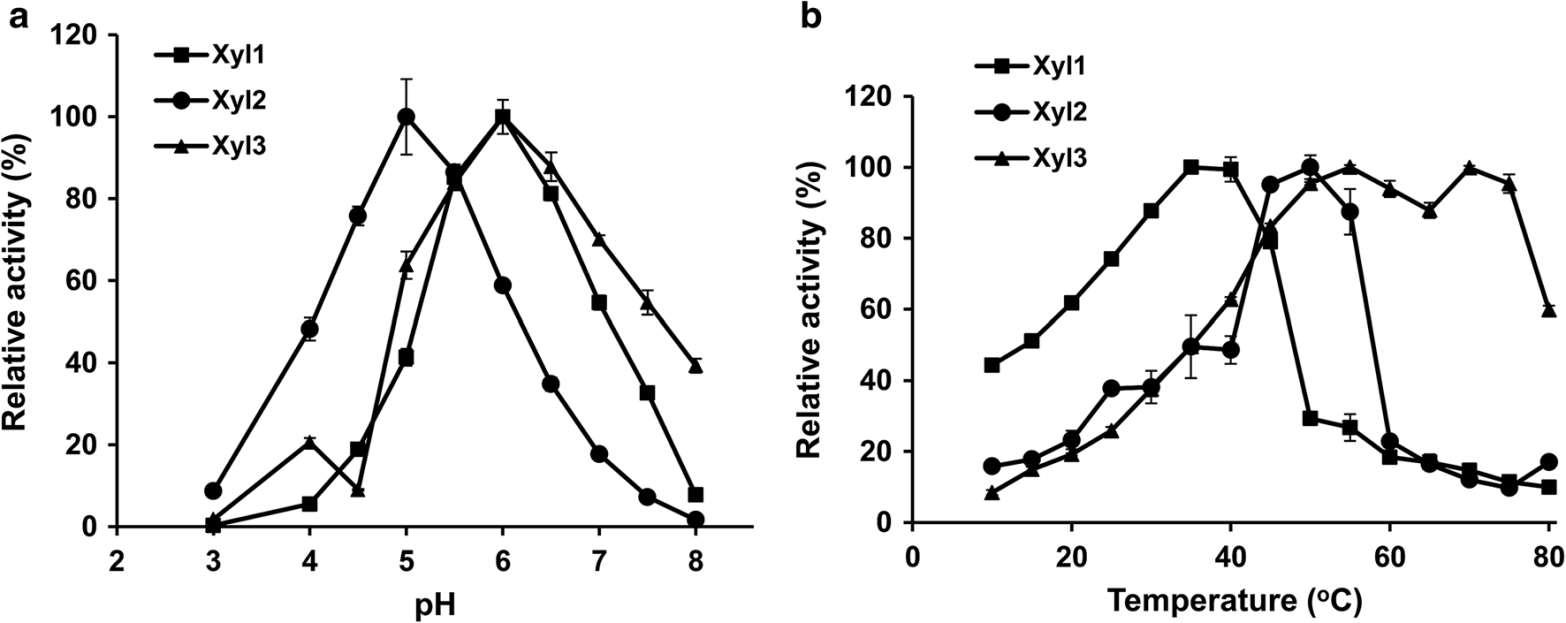
Effects of pH and temperature on the recombinant xylanases. **a** The effect of pH on the activities of Xyl1, Xyl2 and Xyl3; **b** The effect of temperature on the activities of Xyl1, Xyl2 and Xyl3. The experiments were performed in duplicate, and the data are presented as the means ± standard deviations

The optimal pH was 6.0 for both Xyl1 and Xyl3, while for Xyl2 the optimal pH was 5.0 (Fig. 4a). The optimal temperature was 35 °C, 50 °C and 55 °C for Xyl1, Xyl2 and Xyl3, respectively. In addition, Xyl3 showed a relative activity of 99.8% at 70 °C (Fig. 4b). This experiment has been repeated again and the result confirmed the current data. It may be that the thermal activation property of Xyl3 at 70 °C made its activity go down with temperature and then go up. Thermal activation property has been found in *Cb*Man5A [27].

Metal ions have been reported to affect xylanase activities [13]. In order to detect the effect of metal ions on the three xylanases activities, Xyl1, Xyl2, and Xyl3 were incubated with different metal ions (10 mM) for 1 h at 4 °C and the residual enzyme activity was determined. Most of the assayed metal ions exhibited different degrees of inhibition on the activities of the three xylanases, with Ag^+^ and Cu^2+^ showing considerable inhibitory effects (Table 2). These results were similar to those of previously reported xylanases [20, 28, 29]. In contrast, K^+^ and Na^+^ promoted the activities of all three xylanases, making their relative activities in the range of 102.6–109.4%. In addition, Co^2+^, Zn^2+^ and Al^3+^ promoted the activity of Xyl1. The three xylanases could maintain relative activities of greater than 80% in the presence of Zn^2+^, Mg^2+^, Co^2+^ and Ni^2+^. In contrast, the xylanases Xyl-IIb from *P. citrinum* HZN13 [30] and rXyn162 from *Pleurotus ostreatus* HAUCC 162 [31] retained relative activities of only 18% and 56% in the presence of 10 mM Ni^2+^, respectively. In addition, the xylanases xyn10B [20] and XYN11A [32] from *P. oxalicum* GZ-2 retained relative activities of only 37.2% and 67.8% in the presence of 10 mM Co^2+^, respectively.

**Table 2:** Effect of metal ions (10 mM) on the relative activities of the purified recombinant xylanases

Metal ions	Relative activity (%)		
	Xyl1	Xyl2	Xyl3
Control	100.0 ± 0.9	100.0 ± 1.2	100.0 ± 0.5
Ca^2+^	94.3 ± 1.1	80.4 ± 4.8	89.2 ± 0.4
K^+^	109.4 ± 0.6	105.0 ± 1.1	105.3 ± 0.5
Ag^+^	17.5 ± 0.4	8.7 ± 1.0	5.0 ± 0.2
Pb^2+^	78.9 ± 0.3	43.8 ± 0.3	53.5 ± 0.7
Ni^2+^	100.1 ± 0.1	81.6 ± 0.3	98.2 ± 3.0
Mg^2+^	97.2 ± 0.7	84.0 ± 0.1	93.4 ± 1.1
Cu^2+^	54.7 ± 0.7	11.4 ± 0.4	14.2 ± 0.6
Co^2+^	103.6 ± 0.4	82.7 ± 0.6	86.4 ± 0.2
Na^+^	108.9 ± 0.8	102.6 ± 0.3	102.8 ± 1.1
Fe^3+^	95.7 ± 0.3	42.2 ± 0.3	67.2 ± 0.4
Zn^2+^	109.8 ± 0.2	81.7 ± 0.7	96.6 ± 0.2
Al^3+^	108.3 ± 0.2	61.9 ± 2.1	97.4 ± 0.1

Experiments were carried out in the presence of 10 mM metal ions at optimal conditions (35 °C and pH 6.0 for Xyl1; 50 °C and pH 5.0 for Xyl2; 55 °C and pH 6.0 for Xyl3), using 1.0% beechwood xylan as substrate. The relative enzyme activity was calculated using the activity of the enzyme without any added metal ions as 100%

The experiments were performed in duplicate, and the data are presented as the means ± standard deviations

### Synergistic effect of different xylanases on the degradation of delignified corn stover

To investigate the synergistic effect among the three xylanases, Xyl1, Xyl2, Xyl3 alone or in combination were evaluated for the hydrolysis of delignified corn stover. As shown in Table 3, after 48 h of hydrolysis, Xyl1, Xyl2 and Xyl3 released 0.014, 0.033 and 0.345 mg/mL of reducing sugars, respectively. For the four combinations of the three xylanases (Xyl1 + Xyl2, Xyl1 + Xyl3, Xyl2 + Xyl3, and Xyl1 + Xyl2 + Xyl3), the amount of reducing sugars released varied from 0.082 to 0.705 mg/mL, with the Xyl2 + Xyl3 and Xyl1 + Xyl2 + Xyl3 combinations being significantly more effective than the corresponding enzymes alone, and the Xyl1 + Xyl2 + Xyl3 combination was more efficient than the Xyl2 + Xyl3 combination. These results indicated that the three xylanases have synergistic effects, especially between Xyl3 and Xyl2, which belong to different families, whereas the observed synergism between xylanases of the same family (Xyl1 and Xyl3) was not significant (Table 3 and Additional file 2: Table S2).

**Table 3:** Reducing sugars (mg/mL) released by individual and combined of the three wild-type xylanases against delignified corn stover

Enzymes	6 h	12 h	24 h	48 h
Xyl1	0.007 ± 0.001^a^	0.007 ± 0.000^A^	0.002 ± 0.000^α^	0.014 ± 0.001^i^
Xyl2	0.028 ± 0.009^ab^	0.049 ± 0.019^AB^	0.036 ± 0.018^αβ^	0.033 ± 0.013^i^
Xyl3	0.041 ± 0.001^ab^	0.138 ± 0.011^CD^	0.212 ± 0.020^γ^	0.345 ± 0.016^iii^
Xyl1 + Xyl2	0.055 ± 0.025^b^	0.085 ± 0.037^BC^	0.063 ± 0.023^β^	0.082 ± 0.025^ii^
Xyl1 + Xyl3	0.057 ± 0.008^b^	0.153 ± 0.004^D^	0.256 ± 0.019^γ^	0.369 ± 0.004^iii^
Xyl2 + Xyl3	0.327 ± 0.024^c^	0.442 ± 0.034^E^	0.541 ± 0.020^δ^	0.652 ± 0.033^iv^
Xyl1 + Xyl2 + Xyl3	0.345 ± 0.012^c^	0.495 ± 0.015^E^	0.588 ± 0.025^δ^	0.705 ± 0.006^v^

The experiments were performed in triplicate, and the data are presented as the means ± standard deviations

Statistical significance among the data in the same column is indicated by different letters as assessed by ANOVA (Duncan test, *p *< 0.05)

### Synergistic hydrolysis of xylanases and commercial cellulase

**Fig. 5:**
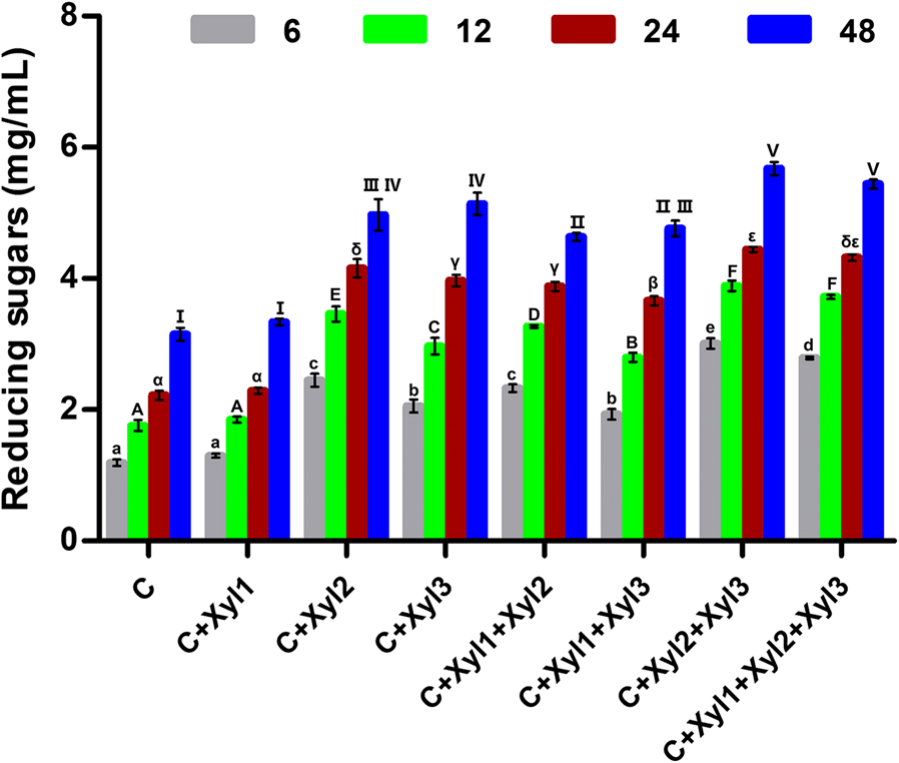
Release of reducing sugars from delignified corn stover by commercial cellulase and the mixture of commercial cellulase and xylanases. C: Commercial cellulase. Gray columns, green columns, red columns and blue columns represent hydrolysis for 6 h, 12 h, 24 h and 48 h, respectively. The experiments were performed in triplicate, and the data are presented as the means ± standard deviations. Statistical significance is indicated by different series of letters in the columns (a,b…for gray columns; A,B…green columns; α,β…for red columns; I,II…for blue columns) as assessed by ANOVA (Duncan test, *p *< 0.05)

Based on previous reports that xylanases can improve the hydrolytic activity of cellulases [5, 33], the potential of Xyl1, Xyl2 and Xyl3 to enhance the hydrolytic performance of a commercial cellulase using delignified corn stover as a substrate was assessed using a supplementation strategy. After 48 h of hydrolysis, the commercial cellulase alone released 3.15 mg/mL reducing sugars (Fig. 5). In contrast, 4.97 and 5.14 mg/mL of reducing sugars were released through the cooperative activities of the commercial cellulase with Xyl2 and Xyl3, significant increases (*p* < 0.05) of 57.6% and 63.1% over that observed using cellulase alone, respectively. In addition, the glucan conversion increased by 44.3% and 46.2%, and the xylan conversion increased by 84.3% and 107.5%, respectively (Additional file 3: Fig. S1). In contrast, Xyl1 did not show a synergistic effect with the commercial cellulase (Fig. 5 and Additional file 3: Fig. S1).

To evaluate the synergistic effects of different xylanases on the hydrolysis of cellulase, different combinations of xylanases [with the total xylanase dosage at 5 mg protein/g glucan in reactions containing two (1:1) or three (1:1:1) xylanases] was supplemented to commercial cellulase (10 mg protein/g glucan) for the hydrolysis of delignified corn stover. In all of these combinations, the combination C + Xyl2 + Xyl3 released the most reducing sugars (5.68 mg/mL) (Fig. 5). The amount of reducing sugars released by C + Xyl1 + Xyl2 + Xyl3 (5.44 mg/mL) was similar to that released by C + Xyl2 + Xyl3, with both combinations releasing significantly greater amounts of reducing sugars than those released by the combinations C + Xyl1 + Xyl2 and C + Xyl1 + Xyl3 (*p* < 0.05). Supplementation of all of the two- and three-enzyme mixture significantly increased the release of reducing sugars compared to that observed using the commercial cellulase alone (*p* < 0.05). Supplementation of cellulase reaction with Xyl2 and Xyl3 resulted the highest level of observed glucan and xylan conversion, 37.3% and 20.8%, representing increases of 49.1% and 121.4% relative to those obtained using the commercial cellulase alone, respectively (Additional file 3: Fig. S1). These results showed that the addition of the xylanases significantly increased the hydrolysis of cellulase, because the hydrolysis of hemicellulose increased the exposure of cellulose to the enzymes.

### Hydrolytic modes of xylanases

**Fig. 6:**
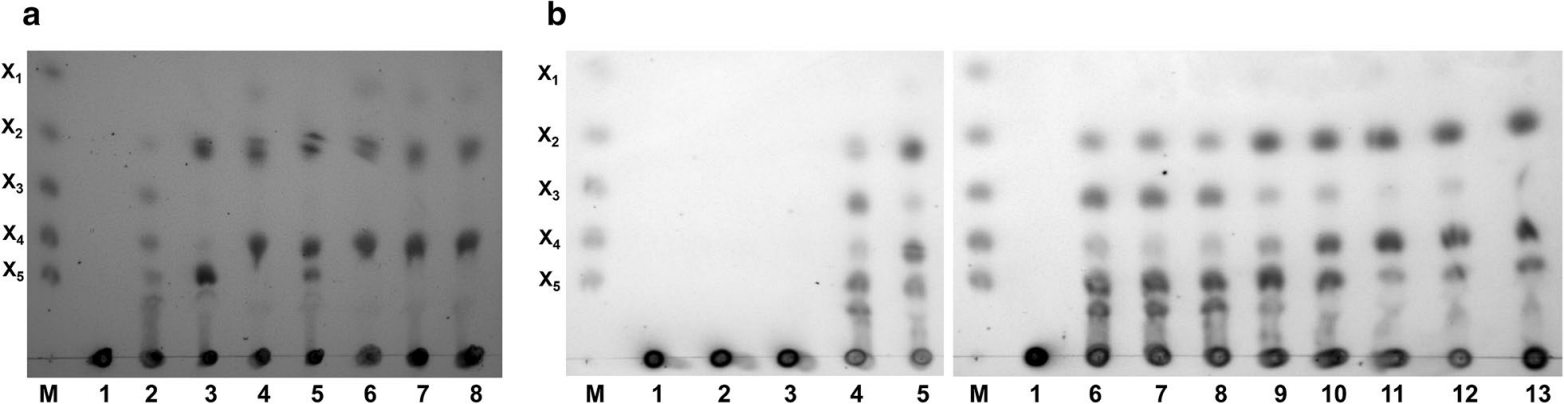
Hydrolysis of beechwood xylan by the recombinant xylanases. **a** Individual and combined enzymatic hydrolysis of beechwood xylan. M, Size markers for xylose (X_1_), xylobiose (X_2_), xylotriose (X_3_), xylotetraose (X_4_) and xylopentaose (X_5_); 1, control no digested samples; 2, Xyl1; 3, Xyl2; 4, Xyl3; 5, Xyl1 + Xyl2; 6, Xyl1 + Xyl3; 7, Xyl2 + Xyl3; 8, and Xyl1 + Xyl2 + Xyl3; **b** Sequential hydrolysis of beechwood xylan. M, Size markers for X_1_, X_2_, X_3_, X_4_ and X_5_; 1, control no digested samples; 2, 6 and 10, hydrolysis with Xyl1, Xyl2 and Xyl3 alone for 2 h, respectively; 3, 4 and 5, hydrolysis with Xyl1 for 2 h following supplementation with Xyl1, Xyl2 and Xyl3 for another 4 h, respectively; 7, 8 and 9, hydrolysis with Xyl2 for 2 h following supplementation with Xyl2, Xyl1 and Xyl3 for another 4 h, respectively; 11, 12 and 13, hydrolysis with Xyl3 for 2 h following supplementation with Xyl3, Xyl1 and Xyl2 for another 4 h, respectively

The synergistic effects between different xylanases have been reported previously, but the results using enzymes from different origins were quite different. To elucidate the mechanism that allows xylanases to exhibit synergism, the hydrolytic modes of the three xylanases toward beechwood xylan were determined. As shown in Fig. 6a, the hydrolytic products produced by Xyl1 were primarily xylotriose, xylotetraose, xylopentaose, unknown xylo-oligosaccharides and a small amount of xylobiose. The major hydrolytic products generated by Xyl2 were xylobiose and xylopentaose, while the hydrolysates produced by Xyl3 were primarily xylobiose, xylotetraose and trace amounts of xylose, indicating that the hydrolytic products generated by Xyl3 had a lower degree of polymerization than those obtained using Xyl2. These results agreed with those of previous reports in which the hydrolytic products released by GH10 xylanases were one Xyl*p* residue shorter than those produced by GH11 xylanases [7, 10]. This result also suggested that the significant synergy between Xyl2 and Xyl3 may result from cooperative hydrolysis modes. Furthermore, the product profiles generated by the combination of the three xylanases using beechwood xylan were also determined. Compared with the hydrolytic products produced by Xyl2 alone, the amount of xylopentaose was reduced and xylotetraose was produced when beechwood xylan was hydrolyzed by the combination of Xyl1 and Xyl2. Furthermore, xylopentaose produced by Xyl2 was completely hydrolyzed into xylotetraose by the combination of Xyl3 and Xyl2, consistent with the results that the combination of Xyl2 and Xyl3 had a superior synergistic effect than the combination of Xyl2 and Xyl1 (Table 3). These results indicate that the different synergistic effects between Xyl1 and Xyl2 or Xyl3 and Xyl2 are primarily associated with the different hydrolytic products that they generate.

The existence of synergistic effects between different xylanases, especially between GH10 and GH11 families, raised the question of what the specific order of action is for the two xylanases in the hydrolysis process. To solve this problem, sequential hydrolysis of beechwood xylan was performed using the three xylanases (Fig. 6b). Due to the low specific activity of Xyl1 toward beechwood xylan (Table 1), no xylooligosaccharide was detected using Xyl1 after 6 h of hydrolysis (Lane 3). However, after the hydrolysis of beechwood xylan with Xyl1 for 2 h followed by Xyl2 for 4 h, the primary observed products were xylobiose, xylotriose, xylotetraose, xylopentaose and xylohexaose (Lane 4). In contrast, the primary observed products when beechwood xylan was hydrolyzed with Xyl1 for 2 h followed by Xyl3 for 4 h were xylobiose, xylotriose, xylotetraose and xylopentaose. Compared to the addition of Xyl2, the use of Xyl3 in cooperation with Xyl1 resulted in a lesser amount of xylotriose and a larger amount of xylobiose (Lane 5). The primary products were xylobiose, xylotriose, xylotetraose, xylopentaose and xylohexaose from beechwood xylan after hydrolysis by Xyl2 for 2 h (Lane 6). The composition and content of the products did not change much even if Xyl2 was added for further hydrolysis for 4 h (Lane 7). However, compared to the product generated by Xyl2 alone, the product profile was altered after the subsequent addition of Xyl3, with a concomitant decrease in the amounts of xylotriose and xylohexaose and an increase in the amount of xylobiose observed (Lane 9). These results indicated that there is synergistic effect between Xyl2 and Xyl3, where the hydrolytic products of Xyl2 are further hydrolyzed by Xyl3 into xylooligosaccharides with a lower degree of polymerization. When beechwood xylan was hydrolyzed by Xyl3 for 6 h, the products were primarily xylobiose, xylotetraose and xylopentaose (Lane 11). After the subsequent addition of Xyl1 or Xyl2, the composition and amounts of the products did not change compared with the hydrolysis by Xyl3 for 6 h (Lane 12 and Lane 13). These results showed that the products of Xyl2 can be further hydrolyzed by Xyl3, but not vice versa. This was the first experiment to demonstrate that hydrolytic products by GH11 xylanases (Xyl2) can be further hydrolyzed by GH10 xylanases (Xyl3) (Lane 9). On the basis of the above results, it can be concluded that the synergy between different xylanases occurs primarily via the cooperative effects of the hydrolysis modes.

**Fig. 7:**
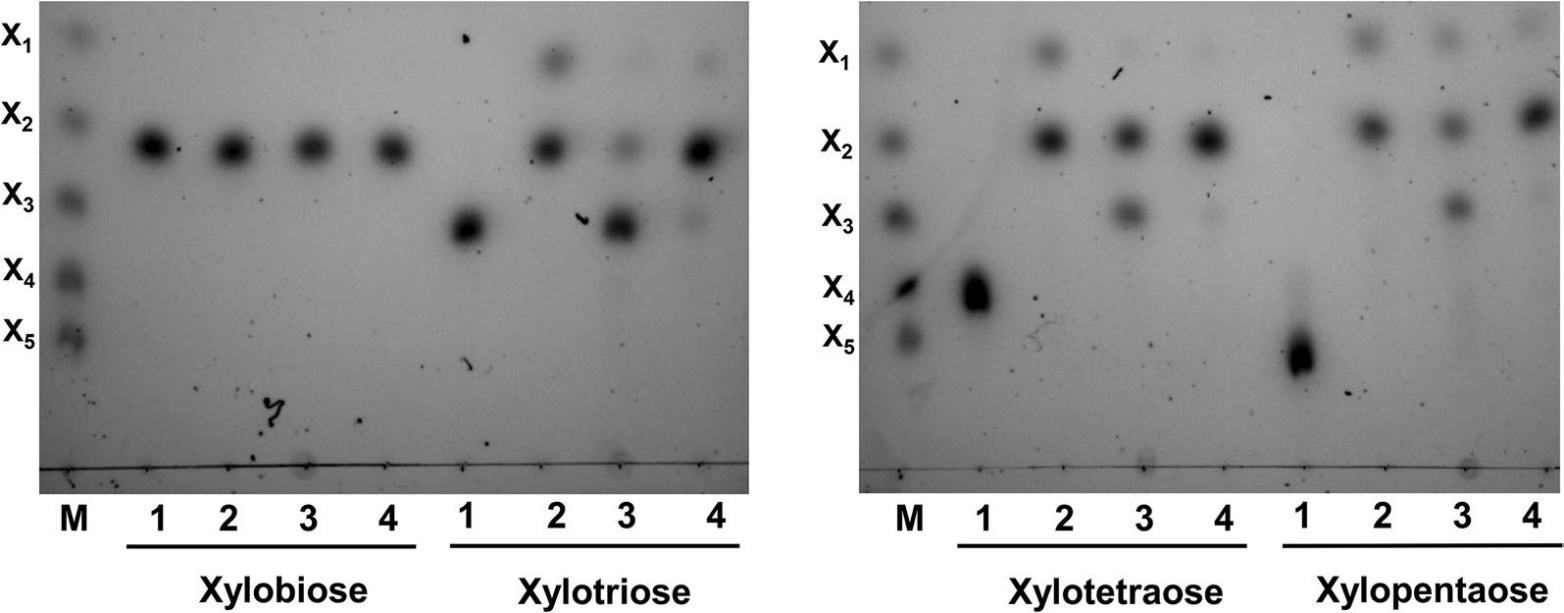
Hydrolysis of xylooligosaccharides by the recombinant xylanases. M, Size markers for X_1_, X_2_, X_3_, X_4_ and X_5_; 1, undigested sample control; 2, Xyl1; 3, Xyl2; 4, Xyl3

With respect to hydrolysis of various xylooligosaccharides, all three xylanases were unable to hydrolyze xylobiose (Fig. 7), which is consistent with previous studies, since most xylanases act in an endo-manner and cannot hydrolyze xylobiose [15, 20, 34, 35]. Xyl2 hydrolyzed xylotetraose and xylopentaose to primarily yield xylobiose, xylotriose and a small quantity of xylose, but it had only weak activity for hydrolyzing xylotriose. Both Xyl1 and Xyl3 could hydrolyze xylotriose, xylotetraose and xylopentaose, but the predominant end products were different. Xylose and xylobiose were produced by Xyl1, but the primary product of Xyl3 was xylobiose.

In order to further detect the difference between Xyl1 and Xyl3, the amount of products released from xylooligosaccharides was also tested by HPLC (Additional file 4: Table S3). Although Xyl1 released less xylobiose than Xyl3, more xylose released by Xyl1. That is to say, although both Xyl1 and Xyl3 are GH10 xylanases, Xyl1 showed lower enzymatic activity toward beechwood xylan and a greater ability to release xylose from xylooligosaccharides than Xyl3. Based on all of the above results, it can be concluded that the three xylanases from *P. chrysogenum* P33 use different strategies and act synergistically to degrade natural xylan. Xyl1 preferentially hydrolyzes xylooligosaccharides, Xyl2 could hydrolyze xylan to long-chain xylooligosaccharides, and Xyl3 hydrolyzed xylan to xylooligosaccharides with a lower degree of polymerization. Thus, an appropriate diversity of xylanases enables microorganisms to use xylan more effectively and exhibit a greater adaptability in complex environments [36, 37].

## Discussion

In order to degrade xylan more efficiently, many microorganisms produced different xylanases in the presence of lignocellulosic materials [11, 16–20]. However, the synergistic mechanism by which different xylanases from the same microorganism promote the degradation of xylan is unclear, which limits the application of xylanases. In our previous study, we found that *P. chrysogenum* P33 secreted three xylanases in the presence of wheat bran plus microcrystalline cellulose [22]. Xyl1 and Xyl3 belong to the GH10 family, whereas Xyl2 belongs to the GH11 family. Besides, Xyl3 possesses a CBM1 domain and an extra loop compared with Xyl1 (Fig. 2). A loop with a similar sequence was previously reported to form a lid above the catalytic site and may play a role in the binding of substrates [16, 38]. Using beechwood xylan as a substrate, the specific activities of Xyl1, Xyl2 and Xyl3 were 151.5 U/mg, 2149.8 U/mg and 290.6 U/mg, respectively (Table 1). The enzymatic activity of xylanases from the same source differed greatly on beechwood xylan [20]. The activity of xylanases derived from fungi was lower than 1000 U/mg in most cases [12, 17]. At present, GH11 xylanases with higher activity were reported to be XylC from *Acremonium cellulolyticus* [39] and XynC from *Talaromyces versatilis* [40], and their activity were 2947 U/mg and 2578 U/mg, respectively. GH10 xylanase with higher activity was reported to be Tlxyn10A from *T. leycettanus*, with specific activity 2240 U/mg [41]. The enzymatic activity of Xyl2 in this study was at a higher level, and Xyl1 and Xyl3 were at a moderate level. These three xylanases were secreted under the same inducing conditions. Therefore, the mechanism of action is worth further study. The optimal temperature/pH values for Xyl1, Xyl2 and Xyl3 were 35 °C/6.0, 50 °C/5.0 and 55 °C/6.0, respectively. The optimum temperature for the reported fungal xylanase was usually 40–60 °C, and the optimum pH was acidic, between 4.0 and 6.0 [42]. However, there are few reports on the properties of all xylanases secreted by the same strain. Xylanases with different optimal reaction conditions that are secreted by a single fungus under the same inducing conditions have been shown to be beneficial to microorganisms by promoting the efficient hydrolysis of lignocellulose in various environments [36, 37].

Although xylanases from different families and/or microorganisms have been reported to act synergistically to hydrolyze lignocellulosic biomass [19, 43], there are few reports on the synergistic effects of xylanases from different families from the same microorganism. In this study, Xyl1, Xyl2, Xyl3 alone and in combination were evaluated for the hydrolysis of delignified corn stover. Synergistic effect exists among different xylanases, and it was higher between Xyl2 and Xyl3, which belong to different xylanase families. And the highest degree of synergism was 4.71 at 6 h (Additional file 2: Table S2). Miao et al. [19] reported that the xylanases Xyn10A and Xyn11A from *Aspergillus fumigatus* acted synergistically in the hydrolysis of washed corncob particles, with the maximum degree of synergism observed of approximately 1.5 at 6 h. Goncalves et al. [43] reported that the xylanases Xyn10B and Xyn11A from *Thermobifida fusca* exhibited the highest degree of synergism at 6 h (3.25) during the hydrolysis of pretreated bagasse. The degree of synergism between Xyl2 and Xyl3 was the greatest degree of synergy between different xylanases from the same microorganism reported to date, demonstrating that the P33 enzyme cocktail is an efficient system to degrade xylan.

In the hydrolysis of beechwood xylan, a higher synergistic effect was observed between Xyl2 and Xyl3 than that between Xyl2 and Xyl1, which was consistent with the hydrolysis of delignified corn stover. Then the mechanism of synergism between Xyl2 and Xyl3 was examined by sequential hydrolysis. The hydrolysates of Xyl2 can be further hydrolyzed by Xyl3 to xylooligosaccharides with lower degree of polymerization. But, the hydrolysis products of Xyl3 can not be hydrolyzed by Xyl2. Until now, the mechanism of synergism between GH10 and GH11 xylanases remains unclear, and our study demonstrated for the first time that the hydrolytic products by GH11 xylanases can be further hydrolyzed by GH10 xylanases, but not vice versa.

At present, the amount of enzyme required to achieve efficient hydrolysis of lignocellulose is still relatively high. Understanding the synergy between xylanases and cellulases to construct lignocellulose-degrading enzyme cocktail is an important way to reduce the dose of enzymes [44]. However, even if various xylanases are used in combination, sometimes it is still difficult to obtain an optimal level of lignocellulose conversion [19, 45]. The primary reason for these results is that the mechanism of action of different xylanases was not clear. Therefore, a thorough study of the hydrolysis modes of different xylanases may help in the selection of appropriate enzymes to develop enzyme cocktails and avoid the use of enzymes with redundant functions, reducing the costs and achieving higher lignocellulose conversion.

## Conclusions

This study described the characteristics and cooperative activities of all three xylanases secreted by *P. chrysogenum* P33. The synergistic effect between Xyl2 and Xyl3 from different families was high, and both could significantly promote the hydrolysis of cellulase. The synergy between different xylanases was primarily due to the cooperative effects of their hydrolysis modes, and this is the first study to demonstrate that the hydrolytic products by GH11 xylanases can be further hydrolyzed by GH10 xylanases, but not vice versa. Cooperation among the three xylanases led to the efficient hydrolysis of hemicellulose. These findings will help us to better understand the mechanism of microbial degradation of hemicellulose.

## Supplementary information

**Additional file 1: Table S1.** All primers used in this study.

**Additional file 2: Table S2.** The degree of synergism between different xylanases.

**Additional file 3: Figure S1.** Conversion of glucan and xylan of delignified corn stover by commercial cellulase and the mixture of commercial cellulase and the recombinant xylanases. C: commercial cellulase. The experiments were performed in triplicate, and the data are presented as the means ± standard deviations. Statistical significance is indicated by different letters in columns as assessed by ANOVA (Duncan test, *p *< 0.05).

**Additional file 4: Table S3.** The amount of products (mg/mL) released by Xyl1 and Xyl3 from xylooligosaccharides.

## Data Availability

All data generated or analysed during this study are included in this published article and its Additional files.

## Abbreviations

CAZy: carbohydrate-active enzyme database
CBM: carbohydrate-binding module
DNS: 3,5-dinitrosalicylic acid
DS: degree of synergism
GH: glycoside hydrolase
HPLC: high-performance liquid chromatography
LB: Luria–Bertani medium
SDS-PAGE: sodium dodecyl sulfate polyacrylamide gel electrophoresis
TLC: thin-layer chromatography
YPD: yeast peptone dextrose medium
