# Supplementary material for: Cooperation of hydrolysis modes among xylanases reveals the mechanism of hemicellulose hydrolysis by Penicillium chrysogenum P33
Source: Microb Cell Fact. 2019 Sep 21;18:159. doi: 10.1186/s12934-019-1212-z (PMC6754857; doi:10.1186/s12934-019-1212-z)
Supplement: Supplementary file 1 — Additional file 1: Table S1. All primers used in this study. [file 12934_2019_1212_MOESM1_ESM.pdf]

Table S1 All primers used in this study

Primer	Sequence (from 5′ to 3′)
Xyl1F	ATGATTCCCAATATCACTCAACTCAAGACAGCT
Xyl1R	CTAGAGAGCAGCTGCGATAGCGTTGTAGGCCTCCTTG
Xyl2F	ATGGTCTCTTTCTCAAGCCTCTTCGTGG
Xyl2R	TTAGGAAACAGTGATGGAAGCAGAT
Xyl3F	ATGGTTCATTTCTCTACCATTGCCCTGGCGTT
Xyl3R	CTATAGACACTGCGAGTACCAGTCATTGATGTGTTT
Xyl1F′	AAGAATTC*CATCATCATCATCATCAT*GGACCCGTTGAATCCCGACAAGCCTCTGAGA
Xyl1R′	AAGGAAAAAAGCGGCCGCCTAGAGAGCAGCTGCGATAGCGTTGTAGG
Xyl2F′	AAGAATTC*CATCATCATCATCATCAT*CTCCCTAATGAGTTGGAGAAGCGGGCCA
Xyl2R′	AAGGAAAATTGCGGCCGCTTAGGAAACAGTGATGGAAGCAGATCCACTGCTCT
Xyl3F′	AAGAATTC*CATCATCATCATCATCAT*GCCGGCTTGCACACCTCTGCTGTTG
Xyl3R′	AAGGAAAAAAGCGGCCGCCTATAGACACTGCGAGTACCAGTCATTGATGTGTTTG

The underlined sequences indicated the recognition sequence of restriction endonuclease *Eco*RI and *Not*I, respectively.

The italic sequences denoted the His tag.
