# Supplementary material for: Cooperation of hydrolysis modes among xylanases reveals the mechanism of hemicellulose hydrolysis by Penicillium chrysogenum P33
Source: Microb Cell Fact. 2019 Sep 21;18:159. doi: 10.1186/s12934-019-1212-z (PMC6754857; doi:10.1186/s12934-019-1212-z)
Supplement: Supplementary file 2 — Additional file 2: Table S2. The degree of synergism between different xylanases. [file 12934_2019_1212_MOESM2_ESM.pdf]

Table S2 The degree of synergism between different xylanases

Enzymes	6 h	12 h	24 h	48 h
Xyl1+Xyl2	1.58	1.54	1.62	1.75
Xyl1+Xyl3	1.18	1.06	1.19	1.03
Xyl2+Xyl3	4.71	2.37	2.18	1.72
