# Supplementary material for: Cooperation of hydrolysis modes among xylanases reveals the mechanism of hemicellulose hydrolysis by Penicillium chrysogenum P33
Source: Microb Cell Fact. 2019 Sep 21;18:159. doi: 10.1186/s12934-019-1212-z (PMC6754857; doi:10.1186/s12934-019-1212-z)
Supplement: Supplementary file 3 — Additional file 3: Figure S1. Conversion of glucan and xylan of delignified corn stover by commercial cellulase and the mixture of commercial cellulase and the recombinant xylanases. C: commercial cellulase. The experiments were performed in triplicate, and the data are presented as the means ± standard deviations. Statistical significance is indicated by different letters in columns as assessed by ANOVA (Duncan test, p < 0.05). [file 12934_2019_1212_MOESM3_ESM.pdf]

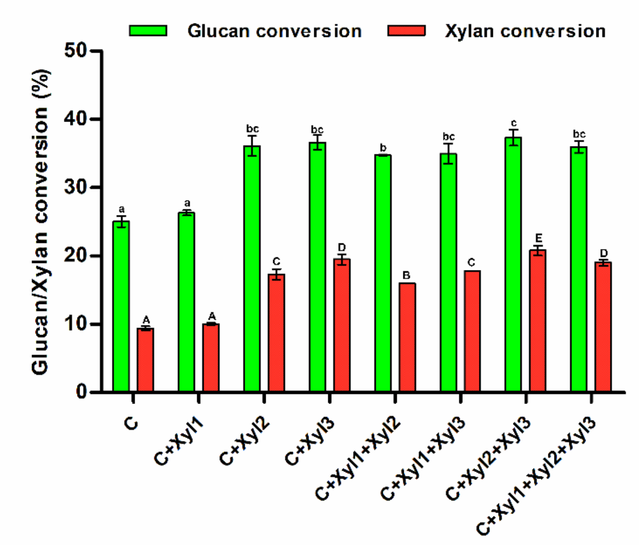

Fig. S1. Conversion of glucan and xylan of delignified corn stover by commercial cellulase and the mixture of commercial cellulase and the recombinant xylanases. C: commercial cellulase. The experiments were performed in triplicate, and the data are presented as the means ± standard deviations. Statistical significance is indicated by different letters in columns as assessed by ANOVA (Duncan test, $p < 0.05$).
