# Supplementary material for: Cooperation of hydrolysis modes among xylanases reveals the mechanism of hemicellulose hydrolysis by Penicillium chrysogenum P33
Source: Microb Cell Fact. 2019 Sep 21;18:159. doi: 10.1186/s12934-019-1212-z (PMC6754857; doi:10.1186/s12934-019-1212-z)
Supplement: Supplementary file 4 — Additional file 4: Table S3. The amount of products (mg/mL) released by Xyl1 and Xyl3 from xylooligosaccharides. [file 12934_2019_1212_MOESM4_ESM.pdf]

Table S3 The amount of products (mg/mL) released by Xyl1 and Xyl3 from xylooligosaccharides

Enzymes	Substrates	X_1	X_2	X_3
Xyl1	X_5	0.107 ±0.008	0.441 ±0.022	0.420 ±0.012
	X_4	0.220 ±0.005	0.520 ±0.012	0.440 ±0.004
	X_3	0.074 ±0.002	0.162 ±0.001	1.075 ±0.019
Xyl3	X_5	0.032 ±0.001	0.748 ±0.028	0.347 ±0.012
	X_4	0.007 ±0.000	0.947 ±0.016	0.325 ±0.009
	X_3	0.017 ±0.001	0.197 ±0.005	1.085 ±0.009

The experiments were performed in triplicate, and the data are presented as the means ± standard deviations.
